# A New Smart 2-Min Mobile Alerting Method for Mild Cognitive Impairment Due to Alzheimer’s Disease in the Community

**DOI:** 10.3390/brainsci13020244

**Published:** 2023-01-31

**Authors:** Yujia Wang, Tong Chen, Chen Wang, Atsushi Ogihara, Xiaowen Ma, Shouqiang Huang, Siyu Zhou, Shuwu Li, Jiakang Liu, Kai Li

**Affiliations:** 1 School of Medical Technology and Information Engineering, Zhejiang Chinese Medical University, Hangzhou 310053, China; 2 Zhejiang-Japan Digital Diagnosis and Treatment and Equipment of Integrated Traditional Chinese Medicine and Western Medicine for Major Brain Diseases Joint Laboratory, Zhejiang Chinese Medical University, Hangzhou 310053, China; 3 Department of Neurology, The Second Medical Center & National Clinical Research Center for Geriatric Diseases, Chinese PLA General Hospital, Beijing 100853, China; 4 Department of Health Sciences and Social Welfare, Faculty of Human Sciences, Waseda University, Tokorozawa 359-1162, Japan; 5 School of Public Health, Hangzhou Normal University, Hangzhou 311121, China

**Keywords:** mild cognitive impairment due to Alzheimer’s disease, spatial execution process, alerting in the community, digital biomarkers

## Abstract

The early identification of mild cognitive impairment (MCI) due to Alzheimer’s disease (AD), in an early stage of AD can expand the AD warning window. We propose a new capability index evaluating the spatial execution process (SEP), which can dynamically evaluate the execution process in the space navigation task. The hypothesis is proposed that there are neurobehavioral differences between normal cognitive (NC) elderly and AD patients with MCI reflected in digital biomarkers captured during SEP. According to this, we designed a new smart 2-min mobile alerting method for MCI due to AD, for community screening. Two digital biomarkers, total mission execution distance ($METR_{total}$) and execution distance above the transverse obstacle ($ED_{above}$), were selected by step-up regression analysis. For the participants with more than 9 years of education, the alerting efficiency of the combination of the two digital biomarkers for MCI due to AD could reach 0.83. This method has the advantages of fast speed, high alerting efficiency, low cost and high intelligence and thus has a high application value for community screening in developing countries. It also provides a new intelligent alerting approach based on the human–computer interaction (HCI) paradigm for MCI due to AD in community screening.

## 1. Introduction

There are about 44 million Alzheimer’s disease (AD) patients worldwide [1], and dementia due to AD accounts for 60% to 70% of all dementia cases [2]; this has become one of the most important medical and social problems [3]. Because there is a long preclinical phase in AD before the onset of the clinical syndrome, the underlying pathophysiological process of AD and its clinical symptomatology are conceptualized as a continuum [4,5]. AD can be divided into three stages [6]: preclinical AD, mild cognitive impairment (MCI) due to AD and dementia due to AD. Among them, preclinical AD [4] is the period during which early AD brain changes are present, but cognitive symptoms have not yet manifested, whereas dementia due to AD [5] is dementia secondary to AD pathophysiology. MCI due to AD [7] is a syndrome defined by clinical, cognitive and functional criteria, which refers to the symptomatic predementia phase of AD. It cannot currently be diagnosed by a laboratory test or the conventional imaging appearances but requires the judgment of clinicians. Clinical and cognitive criteria for MCI [7] include: (1) cognitive concern reflecting a change in cognition reported by the patient, an informant or a clinician, (2) objective evidence of impairment in one or more cognitive domains, typically including memory, (3) preservation of independence in functional abilities, (4) not demented. While MCI due to AD meets the clinical and cognitive criteria of MCI, the etiology of MCI is consistent with the pathophysiological process of AD [7]. In particular, previous studies [8] found that if patients are identified and reasonably treated for MCI due to AD, the reversal rates of MCI patients to normal cognitive function are as high as 30%. Therefore, it is important to identify patients with MCI due to AD.

With the continuous increase in the global elderly population and in human lifespan [9], medical resources in developing countries such as Asia and Africa are clearly insufficient [10,11], which has made alerting the community to MCI due to AD a greater challenge. Currently, early diagnostic methods for MCI due to AD, such as expensive and invasive cerebrospinal fluid examination (CSF) [12], blood biomarker detection [13], positron emission tomography (PET) [14] and magnetic resonance imaging [15] are not suitable for community alerting, while neuropsychological tests represented by mini-mental state examination (MMSE) in the form of paper questionnaires are of great utility in the alert to MCI due to AD in a community [16,17]. However, the application of this approach is limited to large-scale community alerting due to its strong subjectivity, the need for professional evaluation, time consumption, low sensitivity to MCI due to AD [18,19] etc. As a result, it is difficult to meet the needs of large-scale early warning in communities in developing countries such as Asia and Africa. Given the limited scope of primary community alerting, a large number of potential AD patients in the community will lose the opportunity for early intervention. Therefore, it is necessary to find a fast, low-cost and intelligent method for alerting to MCI due to AD.

In order to shorten the screening time to MCI due to AD and improve the convenience of the inspection methods, research has focused on digital biomarkers captured by digital devices during specific tasks designed to alert to MCI due to AD [20,21]. Digital biomarkers are defined as objective, quantifiable, physiological and behavioral elements that are collected and measured through digital devices [22]. Poos Jackie M et al. [23] shortened the test time to 20 min by detecting object position memory and navigation impairment in patients with mild AD dementia and MCI through the Short Digital Spatial Memory Test. In addition, Seixas Azizi et al. [24] extracted a digital neuro signature biomarker that can predict the overall cognitive function and related changes in a 10-min digital cognitive assessment test based on intelligent electronic devices. Koh Tadokoro et al. [25] proposed a novel 3-min eye tracking test, which can effectively distinguish the cognitive function of normal cognitive (NC), MCI and AD subjects. However, the current studies on digital rapid alerts to MCI due to AD still take more than 3 min, and there are generally shortcomings due to the high sample age, the low degree of matching between groups, the low alerting efficacy for MCI due to AD and the need for sensor devices such as eye trackers and electronic pens. Therefore, the real accomplishment of the detection of MCI due to AD at the community, clinical and other large-scale levels still require more in-depth research. A more detailed retrospective analysis is presented in Table 1, including the methodology and limitations of recent studies on MCI alerts.

Abbreviations: NC, normal cognitive; AD, Alzheimer’s disease; MCI, mild cognitive impairment; ROC, the receiver operating characteristic; AUC, the area under the ROC curve. At the same time, the 2.5-min interactive rapid digital human–computer early warning technology [26] we developed earlier took 2.5 min to identify MCI with the aid of an eye tracking device; it had good accuracy in distinguishing between NC elderly and MCI patients, and the area under the receiver operating characteristic (ROC) curve (AUC) reached 0.824. However, the total sample size of the study was limited, including only 32 participants with an average age of 84.5 years. In addition, the technology requires eye tracking devices and game controllers, which are relatively expensive and poorly mobile, making its use difficult for large-scale community alerting. In view of the above shortcomings, we tried to improve the 2.5-min interactive digital human–computer early warning technology to better adapt it to MCI-due-to-AD alerting in densely populated areas such as those in Asia and Africa. 

Although episodic memory impairment is considered to be a typical feature of AD [29], recent studies [30,31,32,33] have suggested that spatial navigation impairment may be another promising cognitive marker of MCI due to AD. As a complex cognitive function, the executive ability of spatial navigation can help individuals design and maintain specific routes, which is essential to the independence, quality of life and safety of older adults [34]. Moreover, the decline in spatial navigation performance is consistent with amyloid and tau deposition and the volumetric declines occurring early in brain regions that subserve navigation [35,36,37]. In a longitudinal study, Verghese et al. [38] evaluated spatial navigation performance to predict the 4-year incidence of MCI in older adults. In addition, cross-sectional studies [39,40] demonstrated the ability of spatial navigation tests to distinguish between older adults with subjective memory problems, MCI and AD. On the other hand, more and more scholars are paying attention to research on evaluating cognition through the executive process. The execution process can be defined as a cognitive mechanism by which performance can be optimized when operating on complex multitasking streams at the same time [41]. Many memory and learning tests involve execution, especially when actions or responses are constantly changing or becoming more complex [42]. P. Allain et al. [43] proved that AD patients have some problems in mentally developing logical strategies and executing complex predetermined plans. These studies on the execution process promoted the transformation of the existing cognitive static evaluation model to the dynamic evaluation model of the whole process and provided a new theoretical basis. 

Therefore, we believe that there is a kind of capability index, the spatial execution process (SEP) index, which can dynamically evaluate the task execution process in spatial navigation task mode. Furthermore, on the basis of this index, this paper proposes the hypothesis that there are neurobehavioral differences between NC individuals and patients with MCI due to AD reflected by digital biomarkers that can be captured during the spatial execution process.

Based on this assumption, a new smart 2-min mobile alerting method was designed for patients with MCI due to AD in the community. This alerting method is suitable for the detection of MCI due to AD in a community with basic education (years of education > 9), and its alerting efficiency can reach 83%, which is higher than that of MMSE (AUC = 0.77). Compared to our previous work [26], the method replaces the eye movement tracking device through fingertip interaction and can be used on mobile tablet devices; this can not only alert faster, but also reduce the economic cost. It can be easily applied for the detection of MCI due to AD in developing countries with a large population base and limited economic and medical resources, such as some Asian and African countries. This method provides a novel intelligent alerting technique based on the paradigm of human–computer interaction (HCI) for the detection of the preclinical stages of AD in the preclinical community.

## 2. Materials and Methods

### 2.1. Participants

In total, 92 participants between 50 and 85 years of age were recruited for the study at a large general hospital in Beijing, including 46 normal cognitive (NC) seniors and 46 patients with MCI due to AD. The study was approved by the ethics committee of Zhejiang Chinese Medical University. All participants volunteered to participate in the experiment and signed an informed consent prior to the experiment.

The NC seniors were recruited according to the criteria that (1) there reported no complaint of cognitive impairment, and their neurological tests were normal, (2) they had a score on the clinical dementia rating scale = 0.5, (3) their daily living ability was normal. Meanwhile, patients with MCI due to AD were recruited according to the 2011 NIA-AA criteria [5,7] i.e., (1) evidence such as CSF or PET showed amyloid β-protein accumulation, (2) their cognitive test scores of one or more cognitive areas were below the norm of 1–1.5 SD typical of individuals of the same age and educational level, (3) they had a score on the clinical dementia rating = 0.5, (4) their capacity for daily living was generally normal.

In addition, exclusion the criteria included: (1) meeting the diagnostic criteria for Parkinson’s disease, frontotemporal dementia, dementia with Lewy bodies or Huntington’s disease, (2) other causes of dementia, such as cerebrovascular disease, central nervous system trauma, etc., (3) a history of schizophrenia, severe anxiety and depression, (4) aphasia, disorders of consciousness and other diseases affecting the cognitive evaluation, tumors, (5) a history of epilepsy or use of antiepileptic drugs, (6) other conditions (e.g., arm disability) that might prevent the completion of the experimental paradigm.

### 2.2. Design of the Paradigm and Experimental Process

Based on the HCI paradigm [26] developed in the previous phase, we designed a mobile rapid alerting paradigm for use in the community. Initially, we set the test time at 150 s based on previous research. However, in the experiment, we found that all NC seniors and almost all MCI patients could complete the test in 2 min. As a result, the test time was ultimately limited to 120 s to meet the screening needs of a large number of people in a community.

MMSE is a classic neuropsychological test that is often used to evaluate the cognitive function of clinical patients and to screen people with cognitive abnormalities in the community [44]. In this study, all participants performed the smart 2-min mobile alerting test and the MMSE to better clarify the alerting efficiency of the smart 2-min mobile alerting method.

**Figure 1:**
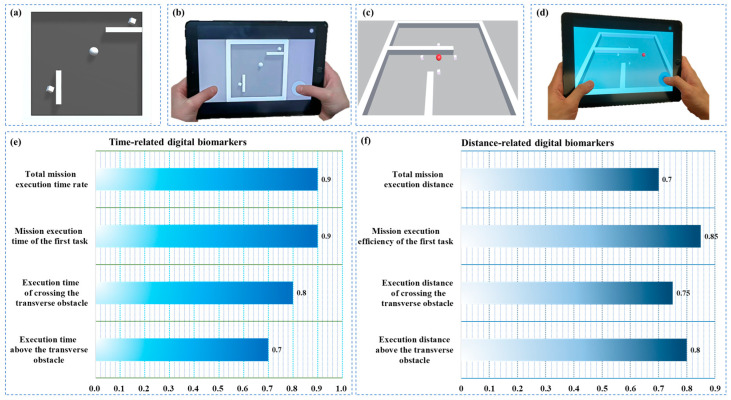
Schematic diagram of (**a**) training level, (**b**) training process, (**c**) 2-min rapid alerting evaluation paradigm of human–computer interaction (HCI), (**d**) evaluation process, (**e**) time-related digital biomarkers selection results, (**f**) distance-related digital biomarkers selection results.

Prior to the formal assessment, the participants would complete a training (not repeated) to become familiar with the paradigm process and manipulation methods. The subjects who could not complete the training were excluded from the group. The teaching level and training process are shown in Figure 1a,b. Spheres, target cubes and obstacles appeared in the formal evaluation scene. The participants needed to use their fingers to manipulate the virtual steering wheel on the tablet to control the sphere, with the task of eliminating all target cubes. The sphere was inert, and the participants needed to predict the motion of the sphere. During the completion of the HCI task, the tablet computer with the relevant system would record all objective operational data of the participants. The 2-min rapid alerting paradigm of HCI is shown in Figure 1c.

### 2.3. Data Acquisition

The device used in this experiment was a tablet computer with a screen resolution of 2560 to 1600 pixels and a size of 10.95 inches. The HCI system, including training module, evaluation paradigm module, and data acquisition module, was designed based on Unity architecture. The HCI data such as sphere coordinate position and order of cube elimination were recorded in the evaluation log file. A schematic diagram of the evaluation process is shown in Figure 1d.

### 2.4. Definition and Quantitative Analysis of Digital Biomarkers

Relevant participant data for the entire paradigm process were collected through digital equipment. Based on the analytic hierarchy process method, eight digital biomarkers were selected and extracted from two dimensions: time and distance [45]. The results are shown in Figure 1e,f. The name and interpretation of the digital biomarkers are shown in Table 2 and Table 3.

In this paradigm, the maximum evaluation time was defined as *T* (s), the total task execution time of the subjects was defined as $T_{e}$ (s), the sample rate was defined as *SR*(Hz), and the maximum cumulative sampling times was defined as $count_{i_{max}}$. The $count_{i_{max}}$ calculation formula is as follows:

(1) $$count_{i_{max}}=T*SR\left(T=120,SR=60\right),$$

When the sampling time point was $T_{i}$, the sphere position was ($X_{Ball_{i}},Y_{Ball_{i}}$), the sphere initial position was ($X_{Ball_{0}},Y_{Ball_{0}}$), and the moving distance of the sphere between the two adjacent sampling points was $d_{i}$. The calculation formula is as follows:

(2) $$d_{i}=\sqrt{X_{B_{i+1}}-X_{B_{i}}{}^{2}+Y_{B_{i+1}}-Y_{B_{i}}{}^{2}}\left(0\leq i\leq count_{i_{max}}-1,i\in N\right),$$

**Figure 2:**
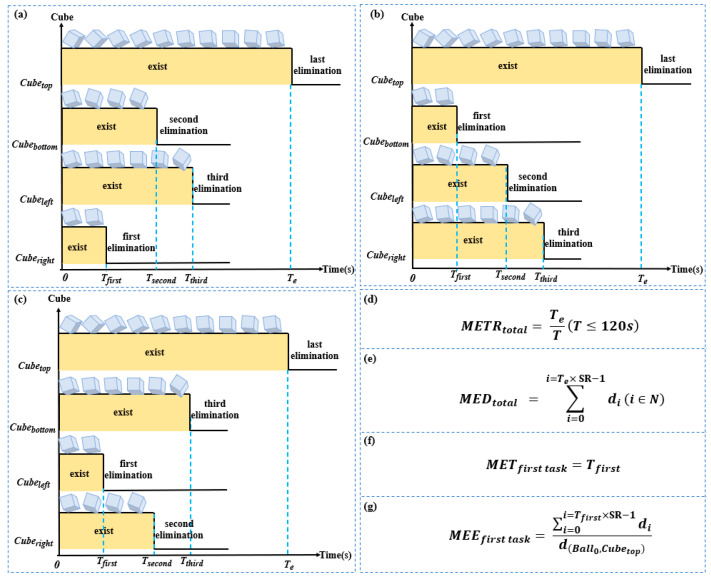
The sequence diagram of eliminating target cubes in different situations and related formulas. (**a**) Sequence diagram of the target cube when eliminating $Cube_{right}$ for the first time, (**b**) sequence diagram of the target cube when eliminating $Cube_{bottom}$ for the first time, (**c**) sequence diagram of the target cube when eliminating $Cube_{left}$ for the first time, (**d**) calculation formula for $METR_{total}$, (**e**) calculation formula for $MED_{total}$, (**f**) calculation formulas for $MET_{first\;task}$, (**g**) calculation formula for $MEE_{first\;task}$.

The target cubes in the four directions of the sphere were $Cube_{top}$, $Cube_{bottom}$, $Cube_{left}$ and $Cube_{right}$. For example, the shortest path distance from $Cube_{left}$ to the sphere was $d_{\left(Ball_{0,}Cube_{left}\right)}$ (px). From a data point of view, $Cube_{top}$ was found not be the first cube eliminated. Thus, the time taken to eliminate the first cube was defined as $T_{first}$. The calculation formulas of $METR_{total}$, $MED_{total}$, $MET_{first\;task}$ and $MEE_{first\;task}$ are showed in Figure 2d–e.

**Figure 3:**
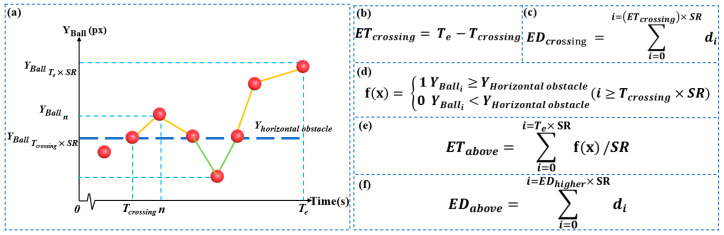
Sequence diagram of the change of the sphere’s Y coordinates and related formulas. (**a**) Sequence diagram of the change of the Y coordinate after the sphere crosses the horizontal obstacle, (**b**) calculation formula for $ET_{crossing}$, (**c**) calculation formula for $ED_{crossing}$, (**d**) discriminant of whether to cross a horizontal obstacle or not, (**e**) calculation formula for $ET_{above}$, (**f**) calculation formula for $ED_{above}$.

At $T_{crossing}$ seconds, the subjects manipulated the sphere over the horizontal obstacle, whose position was ($X_{horizontal\;obstacle},Y_{horizontal\;obstacle}$). The calculation formula for $ET_{crossing}$, $ED_{crossing}$, $ET_{above}$ and $ED_{above}$ are showed in Figure 3b–f.

### 2.5. Statistical Analysis

We used the SPSS 25.0 software package (IBM, Armonk, NY, USA) for statistical analysis. Continuous variables that are not normally distributed are presented as median and quartiles (median [P25, P75]) and compared using the Kruskal–Wallis H rank sum test for differences between groups. Dichotomous variables are expressed as the number of participants and compared using the chi-square test between groups. The value of *p* < 0.05 was considered statistically significant.

## 3. Results

### 3.1. Demographic and Differential Analysis of All Participants

**Figure 4:**
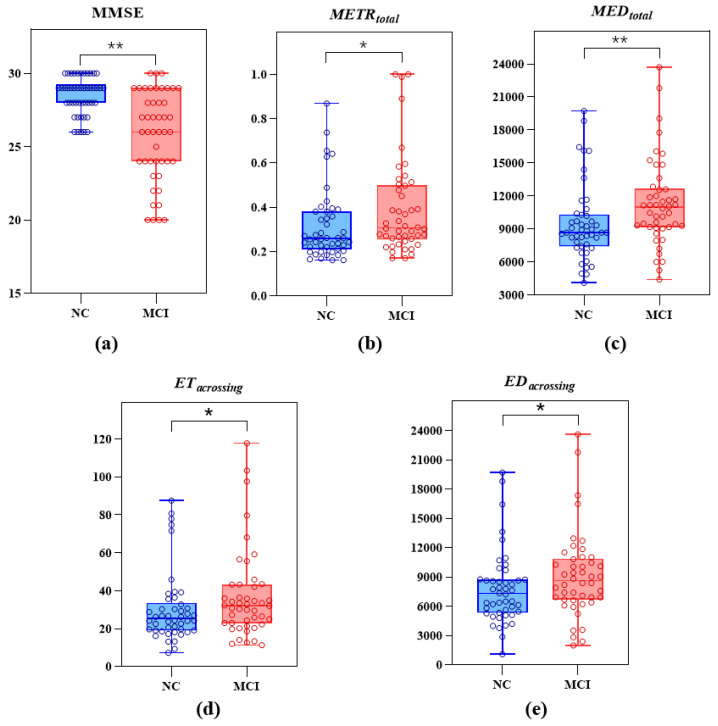
Distribution of (**a**) MMSE data of the NC and MCI groups, (**b**) $METR_{total}$ values of the NC and MCI groups, (**c**) $MED_{total}$ values of the NC and MCI groups, (**d**) $ET_{crossing}$ values of the NC and MCI groups, (**e**) $ED_{crossing}$ values of the NC and MCI groups (* indicates a significant difference between the two groups, at *p* < 0.05; ** indicates a significant difference between the two groups, at *p* < 0.01).

Among the participants, there were 46 NC seniors and 46 patients with MCI due to AD. They were aged 50–85 years. The participants’ demographic data and the results of the differential analysis of the digital biomarkers and MMSE scores between the NC and MCI groups are shown in Table 4; the distribution of the MMSE scores and the values of $METR_{total}$, $MED_{total}$, $ET_{crossing}$, $ED_{crossing}$ of the two groups are shown in Figure 4. On the whole, there were no significant differences in age, sex and years of education between the two groups (*p* > 0.05). In addition, the MMSE scores of the NC group were significantly higher than those of the MCI group (*p* < 0.01), and the $METR_{total}$, $MED_{total}$, $ET_{crossing}$, $ED_{crossing}$ values were significantly lower (*p* < 0.05).

### 3.2. ROC Curves for Identifying MCI Patients from All Participants

**Figure 5:**
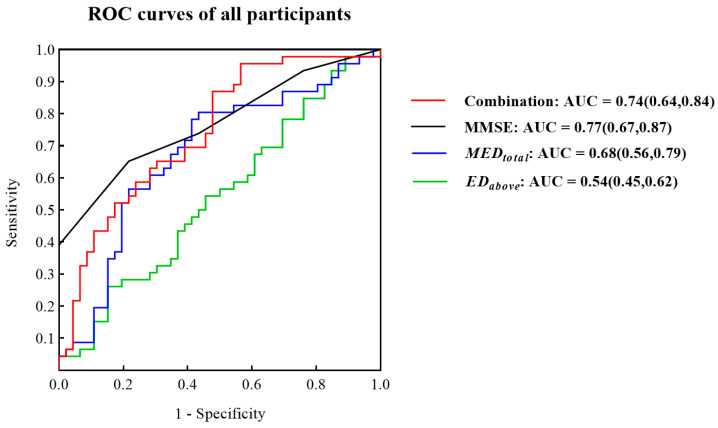
ROC curves of MMSE, $MED_{total}$, $ED_{above}$ and the combination of $MED_{total}$ and $ED_{above}$ for the NC and MCI groups.

Given the small sample size and high dimensions of the eight digital biomarkers, there was the possibility of overfitting of the model. Therefore, we used the stepwise regression method to reduce the dimensions. After dimension reduction, $MED_{total}$ and $ED_{above}$ were retained. As shown in Figure 5, ROC curves suggested that the combination of $MED_{total}$ and $ED_{above}$ could distinguish between NC and MCI but was slightly inferior to the MMSE (Combination AUC = 0.74, MMSE AUC = 0.77).

### 3.3. Demographic and Differential Analysis of the Participants with Basic Education

**Figure 6:**
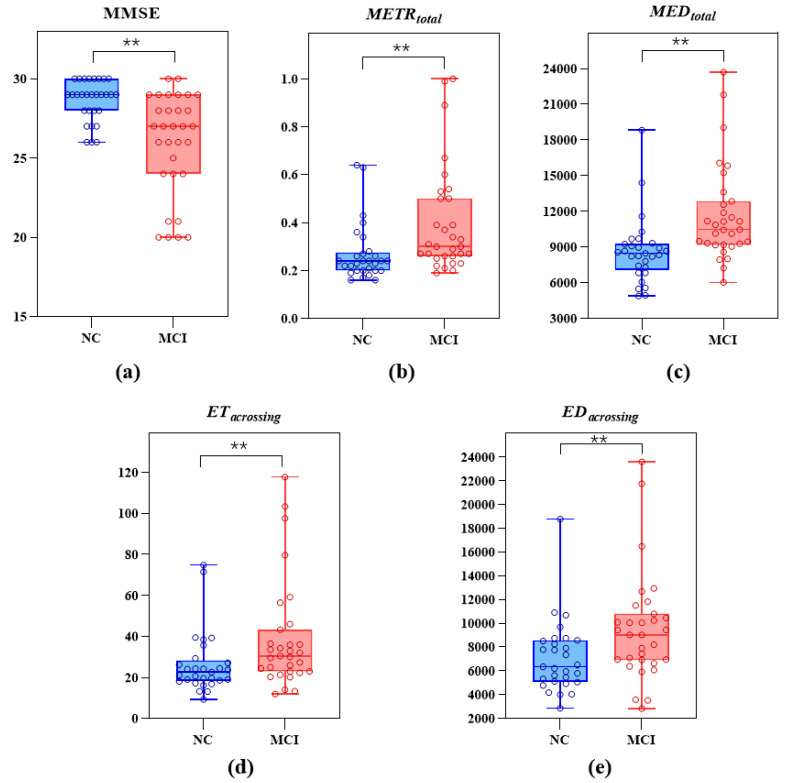
Distribution of (**a**) MMSE scores of the NC and MCI groups with basic education, (**b**) $METR_{total}$ values of the NC and MCI groups with basic education, (**c**) $MED_{total}$ values of the NC and MCI groups with basic education, (**d**) $ET_{crossing}$ values of the NC and MCI groups with basic education, (**e**) $ED_{crossing}$ values of the NC and MCI groups with basic education; (** indicates a significant difference between the two groups, at *p* < 0.01).

Considering the possible impact of the education level on the participants’ use of electronic devices and the fact that China has a 9-year compulsory education system, 29 NC seniors and 31 patients with MCI due to AD (representing 65% of the participants) were included in the NC and MCI groups with basic education. The results from the participants’ demographics and the analysis of the differences in digital biomarkers and MMSE scores between the NC and MCI groups with basic education are shown in Table 5, and the distribution of MMSE scores, $METR_{total}$, $MED_{total}$, $ET_{crossing}$, $ED_{crossing}$ of the participants in both groups are shown in Figure 6. There were no significant differences in age, sex and years of education between the two groups (*p* > 0.05). The MMSE scores for the NC group with basic education were significantly higher than those of the MCI group with basic education (*p* < 0.01), and the values of $METR_{total}$ and $MED_{total}$ were significantly lower (*p* < 0.05).

### 3.4. ROC Curves for Identifying Participants with Basic Education

**Figure 7:**
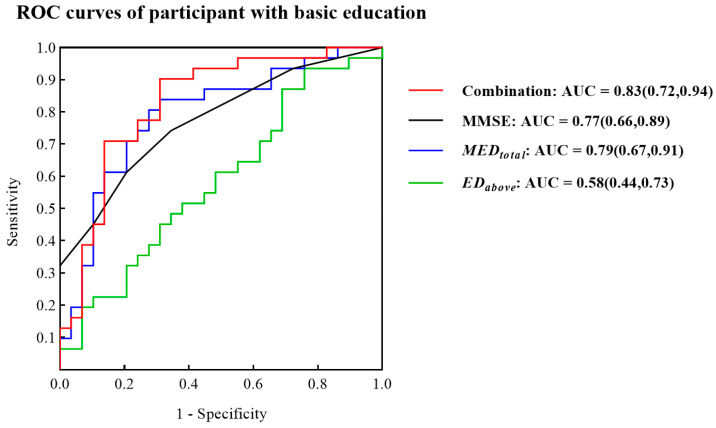
ROC curves of MMSE, $MED_{total}$, $ED_{above}$ and the combination of $MED_{total}$ and $ED_{above}$ for the NC and MCI groups with basic education.

In Figure 7b, the ROC curves showed that $MED_{total}$ allowed a good discrimination (AUC = 0.79) between NC subjects with basic education and patients with MCI due to AD with basic education (years of education > 9) and was better than MMSE (AUC = 0.77). In addition, the AUC of the combination of $MED_{total}$ and $ED_{above}$ was as high as 0.83. 

## 4. Discussion

In this study, four types of digital biomarkers ($METR_{total}$, $MED_{total}$, $ET_{crossing}$, $ED_{crossing}$) measured during the HCI task in patients with MCI due to AD increased significantly, which might be due to an impaired ability of patients with MCI due to AD in spatial navigation [33] and execution process [43]. Based on the dynamic assessment of the cognitive process in the presence of MCI due to AD carried out in this paper, we believe these results suggest integrative SEP impairment, which could be the result of spatial navigation impairment damage to the hippocampus [46,47], or execution processes deficit (including full-process optimal decision planning and fingertip executive dynamic abilities) due to damage to the caudate nucleus [41,42]. In this paper, however, a more thorough distinction was not made between these two possible mechanisms in the examined subjects. These mechanisms will be explored through molecular PET imaging and functional magnetic resonance imaging. In fact, another of our current studies is focused on exploring the mechanisms of the degradation of the HCI execution process caused by damage to the caudate nucleus and cognitive impairment. Compared to the evaluation of visual space and episodic memory function by traditional neuropsychological scales such as the MMSE, the SEP index proposed in this paper focuses more on the objective and dynamic evaluation of the whole cognitive process, which can reflect the ability to comprehensively process individual information, rather than the rigid ability to divide it into different modules. We designed a standardized HCI cognitive evaluation paradigm for “elimination target” tasks, which is mainly based on the SEP index proposed in this paper. It allows the dynamic evaluation of the whole cognitive process time series of visual spatial navigation, path planning and decision making, memory and learning multi-task fusion processing.

In fact, 2 min arethe maximum time considered by the mobile smart alarm method to detect MCI due to AD in a community. The experimental data showed that 92 participants requierd 42.5s on average, and 76 participants could complete the test within 1 min, accounting for 82.6% of the participants. This means that in practical application, the time necessary to the mobile smart alerting method will be further shortened. In addition, the alerting efficiency of this method also reached a high level (AUC = 0.83) for the detection of MCI due to AD in a population with basic education (years of education > 9). At present, many studies have attempted to use digital testing to achieve early and rapid AD warning. Integrated Cognitive Assessment developed by Kalafatis Chris et al. [27] requires 5 min to complete the evaluation and showed an alerting efficiency of 81% for MCI. In addition, the novel eye tracking test developed by Koh Tadokoro et al. [25] took 3 min and was only 75% effective for MCI. Moreover, the Digital Early Warning Technology we proposed earlier [26] took only 2.5 min, and its alerting efficiency reached 82.4% in the study that involved only 32 old participants (average age of 84.5 years). As a new smart 2-min mobile alerting method for MCI due to AD aplicable in communities, the new method shows a significant time cost advantage.

In addition, the smart alerting method only needs a tablet computer for the evaluation, without other auxiliary equipment. In contrast, the digital neuro signature biomarker proposed by Seixas Azizi et al. [24] requires the assistance of multiple sensing devices. In addition, the ROCF developed by Cheah Wen-Ting et al. [28] requires the help of an electronic pen, and the novel eye tracking test developed by Koh Tadokoro et al. [25] and the Digital Early Warning Technology developed by our team [26] both required the assistance of eye tracking devices. In developing countries, such as those in Asia and Africa, additional assistance equipment undoubtedly increases the financial burden of conducting large-scale community screening.

There are also some shortcomings in this study. The first is the small sample size. The number of participants was limited due to the high enrolment requirements. To be enrolled in this study as participants with MCI due to AD, the patients needed a clear diagnosis of AD, including evidence of amyloid β-protein accumulation provided by CSF or PET. In the future, we will conduct research on more people to verify this intelligent alerting method. In addition, the intelligent alerting method here proposed showed poor alert efficacy in the population with low education (years of education ≤9). This may be mainly due to the low familiarity of less educated populations with smart devices, and a simple training proceeding through training levels still is not sufficient to allow them to perform the test skillfully, thus affecting the assessment results. In the future, we will improve the training before the formal evaluation to reduce the impact on the test results of the participants’ different familiarity with the intelligent terminal.

## 5. Conclusions

In summary, we propose a new capability index, the SEP index, which can dynamically evaluate the task execution process in space navigation task mode. Furthermore, on the basis of this index, we hypothesized that there was a neurobehavioral difference between NC subjects and patients with MCI due to AD reflected by digital biomarkers captured during the spatial execution task. Based on this assumption, we designed a new smart 2-min mobile alerting method for MCI due to AD applicable at the community level. Clinical trials have shown that this alerting method is suitable for the detection of MCI due to AD in a community with basic education (years of education >9), and its alerting efficiency can reach 83%. The new smart 2-min mobile alerting method for MCI due to AD for communities has the advantages of fast speed, low cost, high alerting efficiency and intelligence, which will allow an extensive application for community screening of MCI due to AD in developing countries. It also provides a new intelligent alerting approach based on the HCI paradigm for MCI due to AD in community screening.

## Figures and Tables

**Table 1:** Recent studies on methods of digital alerting for MCI and their limitations.

Researcher	Method	Limitation
Li Nan et al. [26]	The 2.5-min interactive rapid digital human–computer early warning technology was developed to accurately distinguish elderly NC patients and MCI patients within 2.5 min, with an AUC of 0.824 by means of an eye tracking device.	(1) The total sample size was limited, (2) only 32 participants were included, and the mean age was 84.5 years, (3) eye tracking equipment and gamepads were required.
Koh Tadokoro et al. [25]	A high-performance eye tracking device was used to analyze the subjects’ eye tracking using the novel eye tracking test and to establish machine learning classification of NC, MCI and AD subjects.	(1) It took 3 min, (2) an eye movement meter was needed, which is expensive, (3) the subjects were old, with an average age of 75 years, (4) ROC curves showed that AUC was only 0.75 for MCI patients.
Kalafatis Chris, et al. [27]	Integrated Cognitive Assessment, a 5-min computerized cognitive assessment tool based on a rapid visual categorization task. It is a rapid visual classification test, which can test the speed of information processing of the subjects and use artificial intelligence to automatically calculate the results of a cognitive impairment screening.	(1) The test time was 5 min, (2) relatively lower recruitment of young subjects with mild AD and mild-AD subjects with high education.
Seixas Azizi, et al. [24]	Sensors such as accelerometers, gyroscopes, magnetic mirrors, cameras, microphones and touch screens capture digital neural biomarkers that can predict the overall cognitive function and changes in older adults with cognitive impairment and cognitive health in a range of motor function tasks and two augmented reality tasks.	(1) The test took 20 min, (2) required multiple sensing devices, (3) there were sample size differences between cognitively impaired and control groups, (4) focused on old adults and lacked an early age group.
Poos Jackie M, et al. [23]	Objective Location Memory Test and Virtual Tubingen Test, such as the Short Digital Spatial Memory Test, were used to examine mild AD dementia and MCI patients.	(1) The test took 20 min, (2) it had a small sample size and age mismatch between groups.
Cheah Wen-Ting, et al. [28]	A digital screening system was designed based on the Rey-Osterrieth Complex Figure neuropsychological test. A tablet and smart pen were also used for data collection to differentiate between MCI and AD patients and healthy controls.	(1) Includes a delayed recall module with a total completion time of 30 min or more, (2) large age differences between sample groups within the dataset, (3) requires an electronic pen.

**Table 2:** Time-related digital biomarkers.

Digital Biomarker	Abbreviation	Unit	Interpretation
Total mission execution time rate	$METR_{total}$	/	Means the ratio of the total execution time of the paradigm to the maximum evaluation time of the paradigm.
Mission execution time of the first task	$MET_{first\;task}$	s	Means how long it takes the participants to eliminate the first target cube.
Execution time of crossing the transverse obstacle	$ET_{crossing}$	s	Means the total amount of time the subject controls the sphere to complete the remaining tasks after crossing the horizontal obstacle in the paradigm scene.
Execution time while above the transverse obstacle	$ET_{above}$	s	Means the total execution time during which the sphere is above the horizontal obstacle in the paradigm scene when the subject controls the sphere to perform the task.

**Table 3:** Distance-related digital biomarkers.

Digital Biomarker	Abbreviation	Unit	Interpretation
Total mission execution distance	$MED_{total}$	px	Means the total distance at which the participants manipulate the movement of the sphere during this paradigm
Mission execution efficiency of the first task	$MEE_{first\;task}$	/	Means the ratio of the distance the subject controls the movement of the sphere to the shortest distance between the sphere and the cube during the time of $MET_{first\;task}$
Execution distance of crossing the transverse obstacle	$ED_{crossing}$	px	Means that the subject controls the sum of the distance the sphere covers after it crosses the horizontal obstacle in the paradigm scene, i.e., the distance the sphere covers during the time of $ET_{crossing}$
Execution distance above the transverse obstacle	$ED_{above}$	px	Means the sum of the distances covered by the sphere while moving over the horizontal obstacle in the paradigm scene when the subject controls the sphere to perform the task, i.e., the distance of movement of the sphere during the time of $ET_{above}$

**Table 4:** Participants’ demographics and differential analysis of digital biomarkers and mini-mental state examination (MMSE) scores between the NC and MCI groups.

	NC *n* = 46	MCI *n* = 46	*p* Value
Age	68.00 (60.75, 79.00)	70.00 (64.75, 80.00)	0.33
Sex (female/male)	29/17	31/15	0.66
Years of education	12.00 (9.00, 15.25)	12.00 (9.00, 14.25)	0.46
MMSE **	29 (28.00, 29.25)	26.00 (24.00, 29.00)	<0.01
$METR_{total}$ *	0.26 (0.21, 0.38)	0.31 (0.25, 0.50)	0.02
$MED_{total}$(px) **	8676.08 (7406.04, 10,324.11)	10,963.08 (9156.78, 12,672.55)	<0.01
$MET_{first\;task}$(s)	1.12 (0.78, 1.75)	1.08 (0.82, 1.96)	0.75
$MEE_{first\;task}$	1.04 (1.01, 1.06)	1.03 (1.01, 1.10)	0.72
$ET_{crossing}$ (s) *	25.32 (19.00, 33.52)	32.18 (22.83, 43.30)	0.04
$ED_{crossing}$(px) *	7292.46 (5331.37, 8750.32)	8606.23 (6675.84, 10,861.47)	0.04
$ET_{above}$(s)	16.51 (12.99, 25.22)	18.27 (13.40, 29.00)	0.54
$ED_{above}$(px)	4497.86 (3567.56, 6036.56)	4279.95 (3508.07, 5780.54)	0.82

Note: two-sample rank sum test with significance of * *p* < 0.05, ** *p* < 0.01.

**Table 5:** Participant demographics and differential analysis of digital biomarkers and MMSE scores between the NC and MCI groups with basic education.

	Basic-Educated NC *n* = 29	Basic-Educated MCI *n* = 31	*p* Value
Age	68.00(61.00, 83.00)	72.00 (64.00, 80.00)	0.62
Sex (female/male)	17/12	17/14	0.77
Years of education	12.00 (9.00, 16.00)	12.00 (6.00, 15.00)	0.08
MMSE **	29 (28.00, 30.00)	27.00 (24.00, 29.00)	<0.01
$METR_{total}\;$*	0.24 (0.20, 0.28)	0.30 (0.26, 0.50)	<0.01
$MED_{total}$(px) **	8469.29 (7071.54, 9285.65)	10,473.75 (9189.11, 12,848.72)	<0.01
$MET_{first\;task}$(s)	0.95 (0.77, 1.56)	1.25 (0.85, 2.30)	0.11
$MEE_{first\;task}$	1.04 (1.01, 1.08)	1.03 (1.01, 1.09)	0.60
$ET_{crossing}$(s)	22.65 (18.37, 28.17)	30.40 (22.98, 43.30)	<0.01
$ED_{crossing}$ (px)	6515.23 (5128.11, 8748.07)	8391.10 (6912.21, 11,023.95)	<0.01
$ET_{above}$(s)	14.03 (12.45, 17.53)	17.72 (12.75, 29.02)	0.26
$ED_{above}$(px)	4040.34 (2928.82, 5167.38)	4260.67 (3579.10, 5640.59)	0.09

Note: two-sample rank sum test with significance of * *p* < 0.05, ** *p* < 0.01.

## Data Availability

Data will be shared upon request.
